# Spontaneous CO Release from Ru^II^(CO)_2_–Protein Complexes in Aqueous Solution, Cells, and Mice**

**DOI:** 10.1002/anie.201409344

**Published:** 2014-12-04

**Authors:** Miguel Chaves-Ferreira, Inês S Albuquerque, Dijana Matak-Vinkovic, Ana C Coelho, Sandra M Carvalho, Lígia M Saraiva, Carlos C Romão, Gonçalo J L Bernardes

**Affiliations:** Instituto de Medicina Molecular, Faculdade de Medicina da Universidade de Lisboa, Av. Prof. Egas Moniz1649-028 Lisboa (Portugal); Department of Chemistry, University of CambridgeLensfield Road, Cambridge CB2 1EW (UK); Instituto de Tecnologia Química e Biológica-António Xavier, Universidade Nova de Lisboa, Av. da República2780-157 Oeiras (Portugal)

**Keywords:** albumin, carbon monoxide, CORM, metalloproteins, prodrugs

## Abstract

We demonstrate that Ru^II^(CO)_2_–protein complexes, formed by the reaction of the hydrolytic decomposition products of [*fac*-RuCl(*κ*^2^-H_2_NCH_2_CO_2_)(CO)_3_] (CORM-3) with histidine residues exposed on the surface of proteins, spontaneously release CO in aqueous solution, cells, and mice. CO release was detected by mass spectrometry (MS) and confocal microscopy using a CO-responsive turn-on fluorescent probe. These findings support our hypothesis that plasma proteins act as CO carriers after in vivo administration of CORM-3. CO released from a synthetic bovine serum albumin (BSA)–Ru^II^(CO)_2_ complex leads to downregulation of the cytokines interleukin (IL)-6, IL-10, and tumor necrosis factor (TNF)-α in cancer cells. Finally, administration of BSA–Ru^II^(CO)_2_ in mice bearing a colon carcinoma tumor results in enhanced CO accumulation at the tumor. Our data suggest the use of Ru^II^(CO)_2_–protein complexes as viable alternatives for the safe and spatially controlled delivery of therapeutic CO in vivo.

Carbon monoxide (CO)-releasing molecules (CORMs) have been shown to mimic the therapeutic effect of gaseous CO in several biological settings.[1,2] Most CORMs described to date are metal carbonyl complexes.[3] Examples include esterase- and phosphatase-triggered Fe-based complexes,[4] photoactivated complexes,[5] vitamin B12 Re-based complexes,[6] or liver-targeted [Mo(CO)_3_(CNR)_3_][7] and [RuCl_2_-thiogalactopyranoside(CO)_3_][8] complexes. However, it has been [*fac*-RuCl(*κ*^2^-H_2_NCH_2_CO_2_)(CO)_3_] (CORM-3) that has attracted most interest due to its therapeutic benefit in animal models of cardiovascular disease, organ transplantation, and acute lung, kidney, and liver injury.[1–2] Nevertheless, there are a number of issues that hinder the clinical development of CORM-3. Its instability in water leads to a half-life in human plasma of only 3.6 min.[9,10] Also, although CORM-3 was initially thought to be a fast CO releaser as assessed by the deoxymyoglobin carbonylation assay, it has been recently demonstrated that it is in fact the reducing agent sodium dithionite used in the assay that promotes the release of CO from CORM-3.[11] This is consistent with the fact that when CORM-3 is dissolved in buffered aqueous solution in a closed vial, only CO_2_ can be detected in the headspace as analyzed by gas chromatography (GC).[10,12,13] This can be explained by a water–gas shift reaction mechanism, in which one CO is first attacked by water resulting in the generation and release of CO_2_ (Figure 1 a).[14] In addition, we[13] and others[15] have shown that complexes of the general formula [RuL_3_(CO)_3_]^2+^ (L=ligand) including CORM-3 react rapidly with proteins to form di- and monocarbonyl Ru^II^–protein adducts. Together, these examples underline the need for a clear understanding of how the in vivo administration of CORM-3 leads to CO release. The elucidation of the CO release profile of CORM-3 in vivo will help guiding the design and synthesis of prodrugs with improved CO release properties and efficacy.

**Figure 1:**
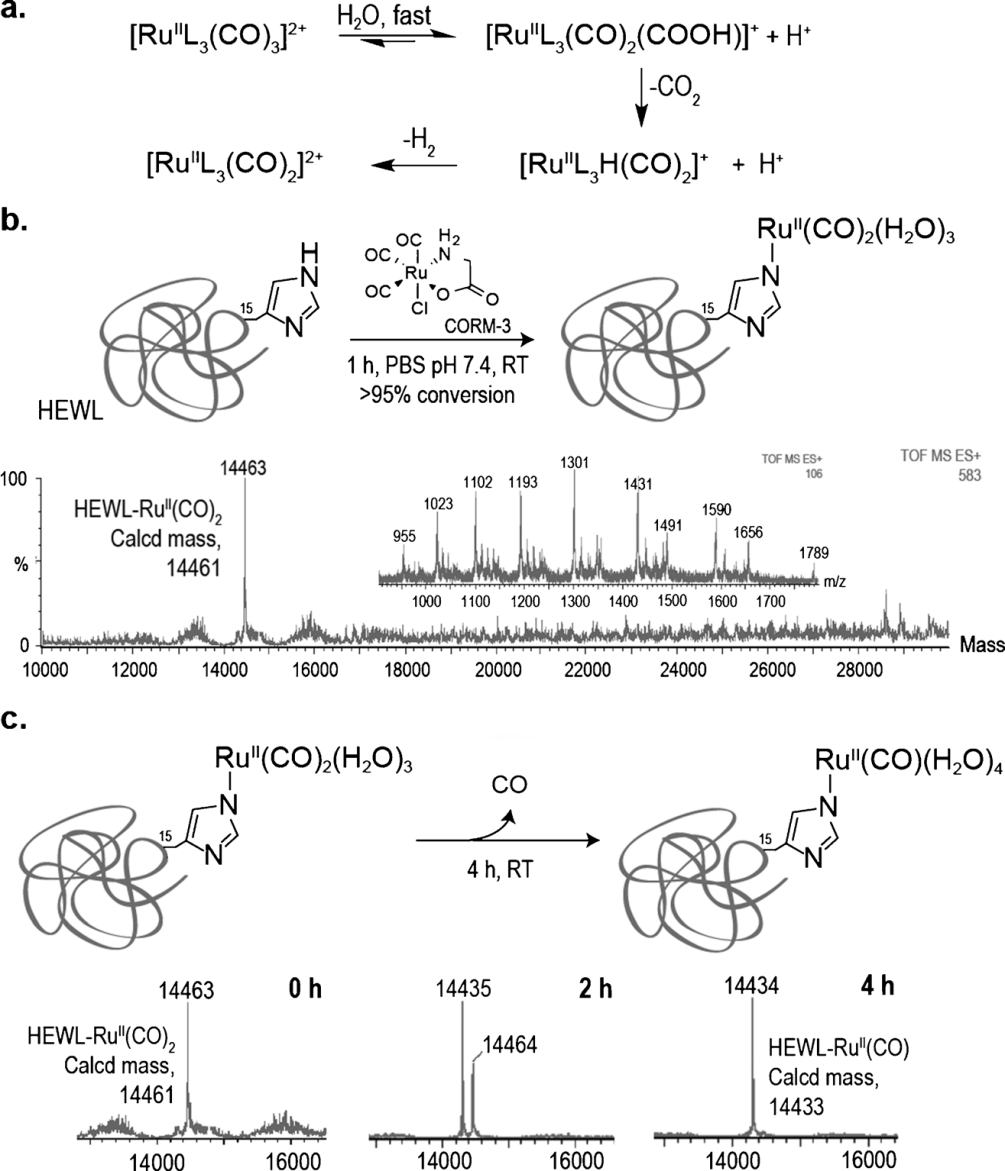
CO release from HEWL–Ru^II^(CO)_2_. a) Reactivity of *fac*-[RuL_3_(CO)_3_]^2+^ complexes in aqueous aerobic solution. b) Reaction of CORM-3 with the single-His protein HEWL and electrospray ionization (ESI)-MS spectrum of HEWL–Ru^II^(CO)_2_ under denaturing conditions. c) ESI-MS analysis of CO release from HEWL–Ru^II^(CO)_2_ at 0, 2, and 4 h in aqueous buffered solution (PBS pH 7.4).

In the present study we set ourselves to elucidate how CORM-3 leads to CO release in vivo. By exploring the chemical instability of CORM-3 in water, we developed a ready method for histidine (His) metalation of proteins. Access to synthetic Ru^II^(CO)_2_–protein complexes allowed to discover that such metalloproteins spontaneously release CO in aqueous solution, cells, and mice. To date, it has remained unclear how exactly the administration of CORM-3 in vivo leads to CO release. In this work, we unequivocally demonstrate for the first time that metalloproteins, formed from the reaction of the hydrolytic decomposition products of CORM-3 with His residues exposed on the surface of proteins, are responsible for the in vivo release of CO and not CORM-3 itself.

We began by building a chemically defined dicarbonyl Ru^II^–protein complex. Access to such a defined protein complex would allow a detailed evaluation of CO release by mass spectrometry (MS) in buffered aqueous solution. We chose hen egg white lysozyme (HEWL) as a model protein due to its single-surface-exposed His at position 15, which has already been shown to react selectively with the [Ru(CO)_2_]^2+^ fragment derived from CORM-3 exposure to water.[13] This fragment is most likely the result of the loss of a chloride ion, a glycinate, and one equivalent of CO_2_, after attack of water at one CO (Figure 1 a). Reaction of HEWL with 50 equivalents of CORM-3 in phosphate-buffered saline (PBS) at pH 7.4 for 1 hour at room temperature produced a single peak with a mass of 14 463 Da corresponding to the successful metalation of HEWL as detected by liquid chromatography MS (LC-MS; Figure 1 b). This is consistent with the capture of [Ru(CO)_2_]^2+^ by His and is in agreement with X-ray diffraction analysis data of HEWL crystals soaked with CORM-3, which showed that the highest occupation site contains the [Ru(CO)_2_]^2+^ fragment bound to His 15.[13] The stability of the purified synthetic HEWL–Ru^II^(CO)_2_ complex was subsequently evaluated in buffered aqueous solution by LC-MS. After 2 h, a new peak with a mass of 14 435 Da could already be observed (Figure 1 c). The new peak, corresponding to the loss of a 28 Da unit of the initial mass of the protein complex, became the main protein peak (>95 % conversion) after 4 h. The loss of a 28 Da unit is consistent with decarbonylation and conversion of HEWL–Ru^II^(CO)_2_ to HEWL–Ru^II^(CO) with consequent release of CO. This result suggests that CORM-3 does not liberate CO spontaneously. Instead, CO is released from the protein complexes formed from the reaction of decomposition products of CORM-3 with His residues exposed on proteins in aqueous solution.

Our MS data, however, does not exclude the hypothesis that the CO lost from HEWL–Ru^II^(CO)_2_ could also be liberated as CO_2_ formed through a water–gas shift reaction (Figure 1 a).[14] Thus, to unequivocally demonstrate that dicarbonyl Ru^II^–protein complexes carry and release CO, we decided to apply the CORM-3 His metalation strategy to albumin as a model carrier protein and use a CO-responsive fluorescent probe to detect CO. Albumin is the most abundant protein in plasma and has been suggested as a possible carrier of CORM-3 fragments in vivo.[13,15,16] We started by modifying bovine serum albumin (BSA) using CORM-3 as a metalation reagent under conditions identical to those previously applied for the His metalation of HEWL. Treatment of BSA with 50 equivalents of CORM-3 at room temperature for 1 hour in PBS pH 7.4 yielded a BSA complex with higher *m*/*z* in >95 % conversion as assessed using nondenaturing nano-electrospray ionization MS (native MS). This corresponds to an average of seven modified His residues from a total of 16 per BSA molecule (Figure 2 a). To detect CO release from the synthesized dicarbonyl Ru^II^–BSA complex we employed the CO-selective turn-on fluorescent probe COP-1 reported by Chang and co-workers.[17] COP-1 selectively reacts with CO through a palladium-mediated carbonylation reaction and was synthesized according to the literature.[17] First, we performed a CO release analysis in buffered aqueous solution at physiological pH (PBS pH 7.4) and were pleased to observe that CO released from the BSA–Ru^II^(CO)_2_ complex triggered a robust fluorescence turn-on response (sevenfold increase within 120 min; Figure 2 b). In the absence of CO, COP-1 is only weakly fluorescent (see Figure S1 in the Supporting Information, SI). Next, we evaluated the ability of the synthetic BSA–Ru^II^(CO)_2_ complex to release CO in HeLa cells using COP-1 to visualize changes of the CO levels by confocal microscopy. HeLa cells were incubated in the absence (control) or presence of 1.5 μm BSA–Ru^II^(CO)_2,_ and then treated with COP-1. Notably, we found a considerable increase in intracellular fluorescence for cells incubated with BSA–Ru^II^(CO)_2_ over the control (Figure 2 c,d). Fluorescence intensity was found to increase in a time-dependent manner (see CO release movie and Figure S2 in SI). Finally, in the presence of BSA–Ru^II^(CO)_2,_ cells remained viable as demonstrated both by the Trypan blue exclusion test (see Figure S3 in SI) and Hoechst 33342 nuclei staining (Figure 2 d).

**Figure 2:**
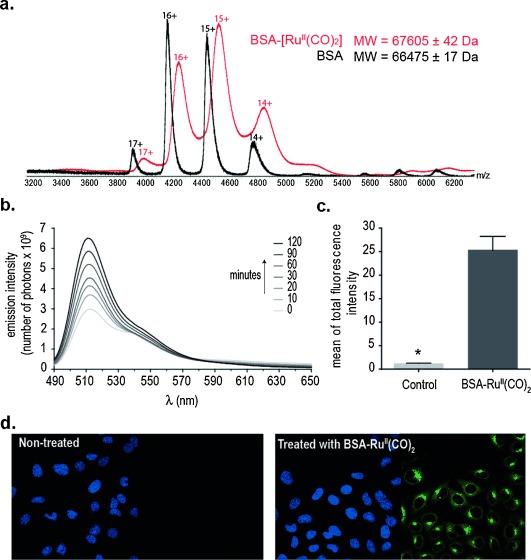
Metalation of BSA with CORM-3 and CO release in buffered aqueous solution and in HeLa cells. a) Native MS of BSA (black) and BSA–Ru^II^(CO)_2_ (red). Peaks for BSA and BSA complex with charges 14+ to 17+ appeared at the 3800 to 5400 *m*/*z* range for both species and there is a shift of peaks for the complex toward higher *m*/*z*. b) CO release measurement using COP-1, read from 490 to 650 nm following excitation (*λ*_ex_=475 nm). Photoemission spectra were taken at 0, 10, 20, 30, 60, 90, and 120 min after the addition of 1 μm COP-1 to 1.5 μm BSA–Ru^II^(CO)_2_ in PBS pH 7.4 at 37 °C. c) Mean of total fluorescent intensity ±standard error of the mean (SEM) in HeLa cells 30 min after addition of 1 μm COP-1 in the absence (control) or presence of 1.5 μm BSA–Ru^II^(CO)_2_. Cells were pre-incubated with BSA–Ru^II^(CO)_2_ for 30 min prior to COP-1 addition. Mean of total fluorescence intensity is given in arbitrary units. Statistically significant differences found after two-way ANOVA are marked as * (P<0,05). d) Confocal microscopy images for cellular CO release in untreated (control) and treated HeLa cells (1.5 μm BSA–Ru^II^(CO)_2_). After an initial 30 min treatment with BSA–Ru^II^(CO)_2_, 1 μm COP-1 was added and, following a 30 min incubation period, pictures were taken. In each panel, the left picture shows nuclear staining using Hoechst 33342 (blue) and the picture to the right shows COP-1 turn-on response to CO (green).

CO and CORMs have been shown to influence the expression levels of cytokines, matrix metalloproteinases, and tumor necrosis factor (TNF)-α.[18–20] To confirm that CO released from the synthetic BSA–Ru^II^(CO)_2_ complex can inhibit inflammatory responses, the expression levels of pro-inflammatory cytokines TNF-α, interleukin (IL)-6, and IL-8, and an anti-inflammatory cytokine, IL-10, were measured by an enzyme-linked immunosorbent assay (ELISA). Treatment of the adenocarcinoma cell line HeLa (Figure 3, graph a) and Caco-2 cells (Figure 3, graph b) with 4.5 μm BSA–Ru^II^(CO)_2_ for 4 h mediated the downregulation of TNF-α, IL-6, and IL-10 in both cell lines (Figure 3 and Figure S4 in SI). IL-8 had low expression levels in both cell lines and no significant difference could be detected upon treatment with BSA–Ru^II^(CO)_2_. Collectively our data demonstrate that Ru^II^(CO)_2_–protein complexes can carry and release biologically functional CO in a time-dependent manner in aqueous solution, Caco-2, and HeLa cells.

**Figure 3:**
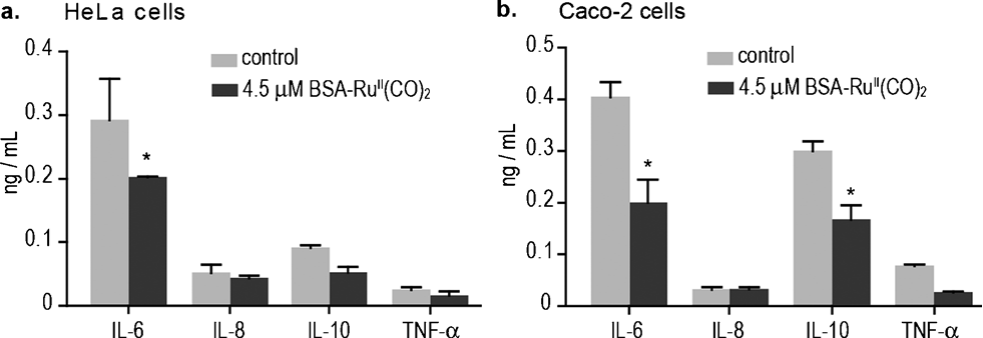
Effect of CO release from BSA–Ru^II^(CO)_2_ on the expression levels of IL-6, IL-8, IL-10, and TNF-α in supernatant of adenocarcinoma cell lines HeLa (graph a) and Caco-2 cells (graph b), measured by ELISA. Cytokine expression was measured 4 h following treatment with 4.5 μm BSA–Ru^II^(CO)_2_ (dark grey) and are presented side by side against the untreated control (light grey). Statistically significant differences found after two-way ANOVA post-hoc test using the Bonferroni method are marked as * (P<0,05).

The potent bactericidal killing activity of CORM-3, which was initially thought to be CO-mediated, has been recently attributed to the generation of reactive oxygen species (ROS).[21] Thus, we decided to perform a direct comparison of the antibacterial activity of CORM-3 and BSA–Ru^II^(CO)_2_ in *E. coli* to assess whether this would be the case for the new CO-releasing metalloprotein. We observed that both unmodified BSA (control) and the CO-releasing BSA–Ru^II^(CO)_2_ complex did not produce any significant effect on bacterial growth (see Figure S5 in SI). These results contrast with those elicited by CORM-3 and indicate that the new BSA–Ru^II^(CO)_2_ complex does not result in the production of ROS species. Importantly, our data suggests that the biological effects of the BSA–Ru^II^(CO)_2_ complex may be assigned to the released CO.

Finally, we investigated whether metalated proteins could be used to carry and deliver CO in vivo to sites of disease by performing a CO biodistribution study in tumor bearing mice. Albumin is a protein known to accumulate in and be catabolized by tumors and has been successfully used for the delivery of drugs to tumors.[22] Balb/C athymic nude mice were inoculated with subcutaneous murine CT-26 colon carcinoma cells and, after tumors reached a volume of 200 mm^3^, were injected intravenously with 3 mg kg^−1^ of BSA–Ru^II^(CO)_2_ or BSA alone (control). Mice were sacrificed 4 h after injection and CO levels were determined in tissues and blood using the method described by Vreman and co-workers.[23] We found that albumin can carry and deliver CO in vivo with selective accumulation at the tumor (Figure 4). Four hours after administration of BSA–Ru^II^(CO)_2_, CO levels at the tumor are threefold higher compared to that of the blood. It must be considered that the method used for measuring CO in tissues and blood does not distinguish between free CO, the synthetic BSA–Ru^II^(CO)_2_ complex and CO bound to hemoglobin, which may account for the relatively high levels of CO found in blood. We have, however, measured the levels of carboxy hemoglobin (COHb) in blood, which were found to be at basal levels (see Figure S6 in SI) excluding any potential CO-associated toxicity. CO accumulation at the tumor is sevenfold higher compared to the kidney and liver, and 17 fold higher when compared to other tissues. By performing a CO in vivo biodistribution measurement, we were able to show that albumin is an efficient CO carrier and specifically delivers CO to tumor sites (Figure 4).

**Figure 4:**
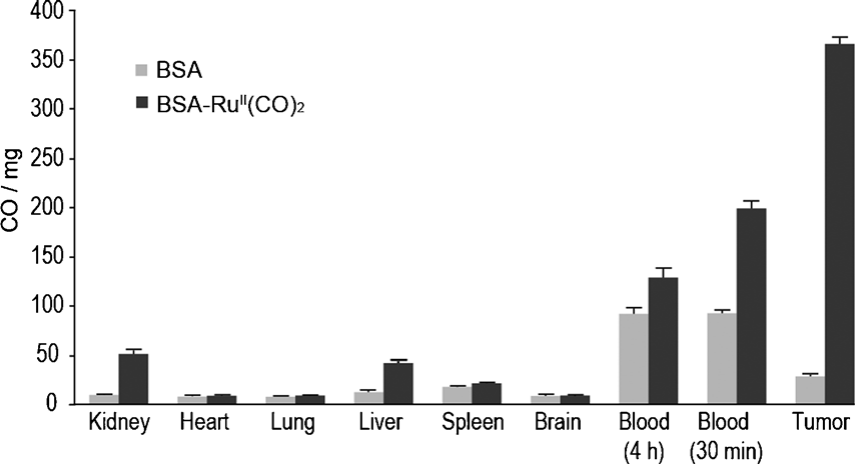
CO biodistribution in blood and tissues after administration of BSA–Ru^II^(CO)_2_ in CT26 colon carcinoma bearing mice. Immunodeficient female BALB/c mice bearing subcutaneous CT26 colon carcinoma cells were administered intravenously (tail vein) with 3 mg kg^−1^ of BSA (light grey) and BSA–Ru^II^(CO)_2_ (dark grey) (three mice/group). Treatment was initiated when tumors reached a size of 200 mm^3^. The animals were sacrificed 4 h after injection. Selected tissues were isolated, homogenized, weighted, and CO levels were measured by gas chromatography reduced compound detection (GC-RCP). Data represents pmol of CO per weighted tissue or organ as a mean of three independent measurements (±standard error). An additional measurement 30 min after injection was performed using collected blood.

In this study we were able to demonstrate for the first time that Ru^II^(CO)_2_–protein complexes, formed by the reaction of decomposition products of CORM-3 with His residues exposed on proteins in aqueous solution, spontaneously release CO in aqueous solution, cells, and mice. Our data provides insight into the mechanism by which CORM-3 leads to CO release in vivo supporting the principle of plasma proteins as in vivo carriers of CO. By exploring CORM-3 reactivity in water, we developed and applied a ready His metalation method that provides access to synthetic Ru^II^(CO)_2_–protein complexes. These were shown to release CO and to influence the inhibition of inflammatory responses as shown by the downregulation of IL-6, IL-10, and TNF-α. Notably, by using a synthetic Ru^II^(CO)_2_–albumin complex we were able to deliver CO specifically to tumors in mice with only basal levels of COHb. This is consistent with the transport of CO through circulation by the Ru^II^(CO)_2_–albumin complex and specific release at the tumor site upon accumulation. Collectively, our data not only provide new insight on how the in vivo administration of CORM-3 results in CO release but also suggests the use of Ru^II^(CO)_2_–protein complexes as viable alternatives for the safe and spatially controlled delivery of therapeutic CO.
