# Supplementary material for: Spontaneous CO Release from RuII(CO)2–Protein Complexes in Aqueous Solution, Cells, and Mice
Source: Angew Chem Int Ed Engl. 2014 Dec 4;54(4):1172–5. doi: 10.1002/anie.201409344 (PMC4506567; doi:10.1002/anie.201409344)
Supplement: Supplementary file 2 [file anie0054-1172-sd2.pdf]

Angewandte Chemie
Eine Zeitschrift der Gesellschaft Deutscher Chemiker

Supporting Information
© Wiley-VCH 2015
69451 Weinheim, Germany

Spontaneous CO Release from $Ru^{II}(CO)_2$–Protein Complexes in Aqueous Solution, Cells, and Mice**

*Miguel Chaves-Ferreira, Inês S. Albuquerque, Dijana Matak-Vinkovic, Ana C. Coelho, Sandra M. Carvalho, Lígia M. Saraiva, Carlos C. Romão, and Gonçalo J. L. Bernardes**

anie_201409344_sm_miscellaneous_information.pdf
anie_201409344_sm_CO_release_movie.avi

Supporting Information

Table of contents

1. Supporting Results

Supporting Figure 1 S3

Supporting Figure 2 S4

Supporting Figure 3 S5

Supporting Figure 4 S6

Supporting Figure 5 S7

Supporting Figure 6 S8

2. Methods

2.1 General procedure for chemical His metallation of proteins using CORM-3 S9

2.2 Protein sequences and expected modifications S9

2.3 Liquid chromatography-mass spectrometry (LC-MS) under denaturing conditions S10

2.4 Nondenaturing Nanoelectrospray Ionization Mass Spectrometry (Native Mass Spectrometry) S10

2.5 Cell culture S11

2.6 Cell viability assay S11

2.7 COP-1 fluorescence response to CO measured in buffered aqueous solution S11

2.8 COP-1 fluorescence response by confocal microscopy imaging S12

2.9 Chemokine modulation by CO release S12

2.10 Bacterial strains and growth conditions S13

2.11 In vivo CO biodistribution in tumor bearing mice S14

2.12 Determination of COHb levels in blood S14

3. References S15

1. Supplementary Results

Supporting Figure 1

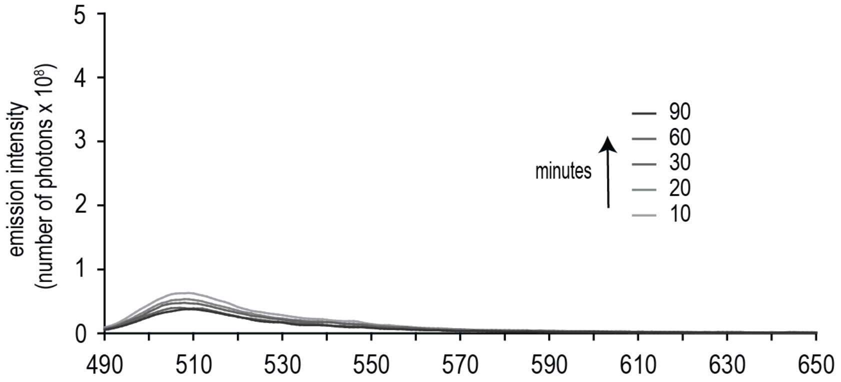

Figure S1. Fluorescence measurement of COP-1 probe in buffered aqueous solution, read from 490 to 650 nm, following excitation (λex = 475 nm). Photoemission spectra were taken at 10, 20, 30, 60 and 90 after the addition of 1 μM COP-1 in PBS pH 7.4 at 37 ºC.

Supporting Figure 2

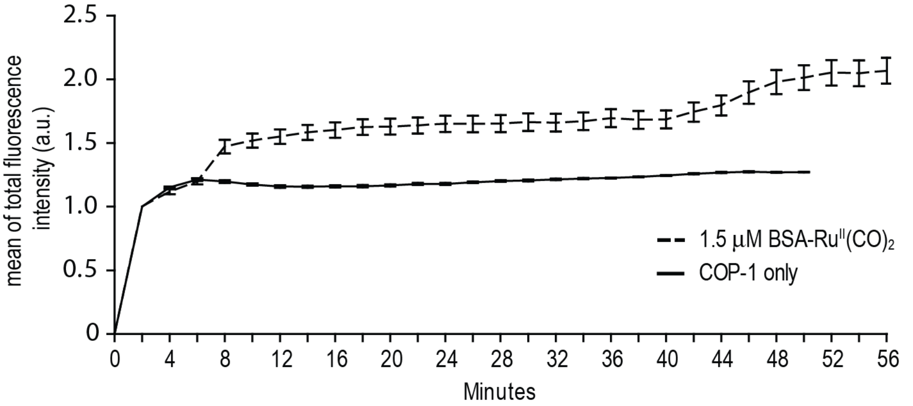

Figure S2. Comparison of mean of total fluorescence intensity ± SEM in arbitrary units (a.u.) of HeLa cells after addition of 1 μM COP-1 in the absence (control) or presence of 0.5 μM BSA-$Ru^{II}(CO)_2$ (pre-incubated for 30 min). Images were taken every two min using representative images of three independent experiments on a per cell basis.

Supporting Figure 3

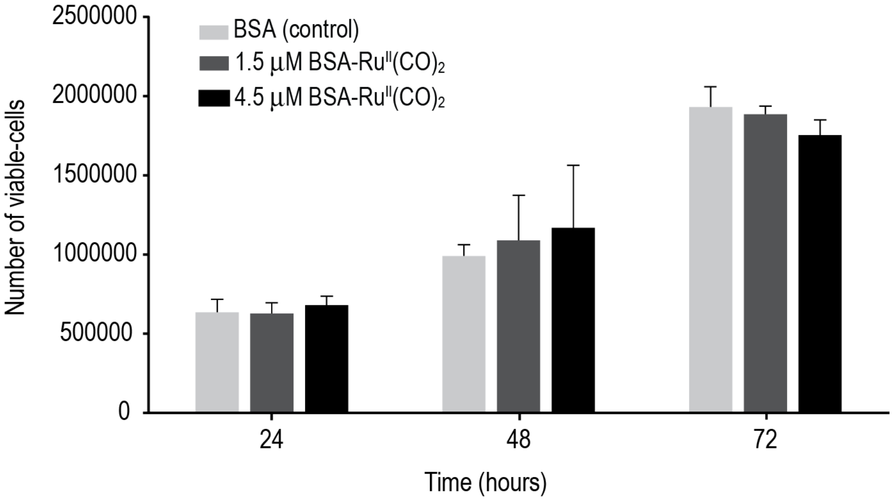

Figure S3. Effects of 24, 48 or 72 hours of incubation with BSA-Ru^{II}(CO)$_2$ in HeLa cells viability. Cells were trypsinized and re-suspended in fresh medium. Cell-numbers were counted in the presence of Trypan blue dye using a Neubauer chamber. Bars represent mean ± SD of a single experiment.

Supporting Figure 4

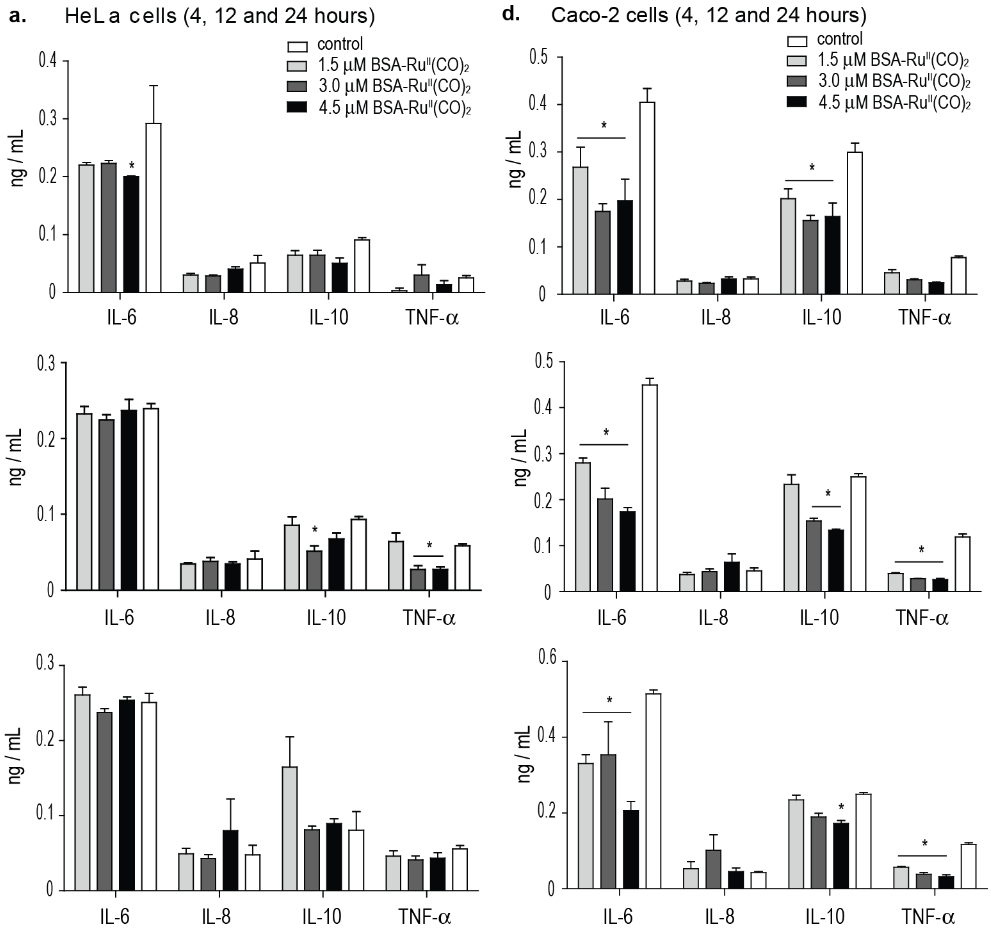

Figure S4. Effect of CO release from BSA-$Ru^{II}(CO)_2$ on the expression levels of IL-6, IL-8, IL-10 and TNF-α in supernatant of adenocarcinoma cell lines HeLa (graph on the left) and Caco-2 cells (graph on the right), measured by ELISA. Cytokine expression was measured 4, 12 and 24 hours, respectively from top to bottom, following treatment with three different concentrations (1.5, 3 and 4.5 μM) of BSA-$Ru^{II}(CO)_2$ (dark grey) and are presented side by side against the untreated control (light grey). Statistically significant differences found after two-way ANOVA post-hoc test using Bonferroni method are marked as * (P<0,05).

Supporting Figure 5

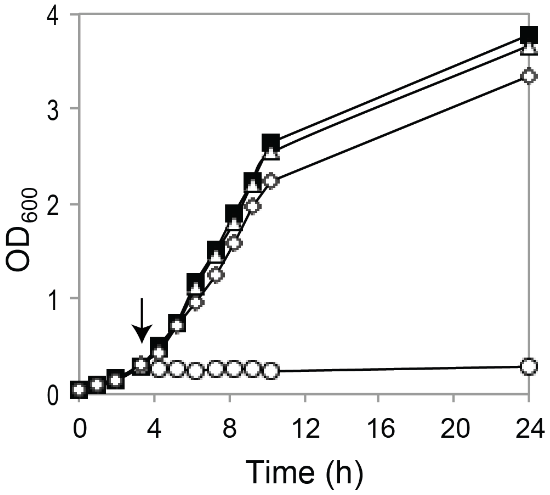

Figure S5. Effect of BSA-$Ru^{II}(CO)_2$ on *E. coli* growth. *E. coli* cells were grown in minimal medium under aerobic conditions to an optical density at 600 nm (OD_{600}) of 0.3 (time-point indicated by the arrow) were left untreated (■) or exposed to 5 µM BSA (△), 50 µM CORM-3 (○) and 5 µM BSA-$Ru^{II}(CO)_2$ (◇). The amount BSA-$Ru^{II}(CO)_2$ was calculated taking into account that each BSA molecule has 7 $Ru^{II}(CO)_2$ fragments attached. Growth curves are representative of data from three biological samples.

Supporting Figure 6

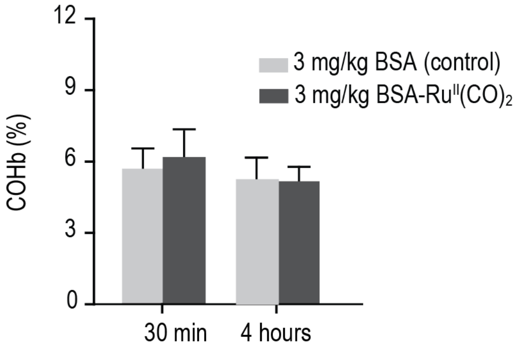

Figure S6. Determination of COHb in the form of percentage of total amount of hemoglobin in blood collected from mice at 30 min and 4 hours after intravenous administration of 3 mg/kg of BSA (control) or BSA-Ru^{II}(CO)$_2$. Groups were composed of 3 mice, and two blood samples were obtained from each mouse.

2. Methods

2.1 General procedure for chemical His metallation of proteins using CORM-3

Proteins used in this study: Hen Egg-White Lysozyme (HEWL) and bovine serum albumin (BSA) were purchased from Sigma Aldrich. Typically, HEWL and BSA solutions were prepared as 1 mg/mL solution in PBS pH 7.4. CORM-3 (50 equivalents) (Sigma-Aldrich) is added as a solid to the protein solution (1 mL, c = 1.0 mg/mL) in PBS pH 7.4 in a plastic tube and the mixture vortexed to homogenize. The reaction is left standing for 30 min at room temperature. Purification of the metallated proteins is achieved by size exclusion chromatography using a HiTrap desalting column (GE Healthcare) to remove excess reagents. Purified samples were used for mass spectrometry analysis using the conditions described in 2.3 and 2.4.

2.2 Protein sequences and expected modifications

Hen Egg White Lysozyme (HEWL) – 1 single Histidine residue

MRSLLILVLCFLPLAALGKVFGRCELAAAMKRHGLDNYRGYSLGNWVCAA
KFESNFNTQATNRNTDGSTDYGILQINSRWWCNDGRTPGSRNLCNIPCSA
LLSSDITASVNCAKKIVSDGNGMNAWVAWRNRCKGTDVQAWIRGCRL

Calculated Isotopically Averaged Molecular Weight of HEWL[1]: 14305.1 Da

Bovine serum albumin (BSA) – 16 Histidine residues

DTHKSEIAHRFKDLGEEHFKGLVLIAFSQYLQQCPFDEHVKLVNELTEFAKTC
VADESHAGCEKSLHTLFGDELCKVASLRETYGD
MADCCEKQEERNECFLSHK DDSPDLPKLK PDPNTLCDEF KADEKKFWGK
YLYEIARR17PYFYAPELLYYANKYNGVFQE CCQAEDKGACLLPKIETMRE
KVLASSARQRLRCASIQKFGERALKAWSVARLSQKFPKAEFVEVTKLVTD
LTKVHKECCHGDLLECADDRADLAKYICDNQDTISSKLKECCDKPLLEKS
HCIAEVEKDAIPENLPPLTADFAEDKDVCKNYQEAKDAFLGSFLYEYSRR
HPEYAVSVLLRLAKEYEATLEECCAKDDPHACYSTVFDKLKHLVDEPQNL
IKQNCDQFEKLGEYGFQNALIVRYTRKVPQVSTPTLVEVSRSLGKVGTRC
CTKPESERMPCTEDYLSLILNRLCVLHEKTPVSEKVTKCCTESLVNRRPC
FSALTPDETYVPKAFDEKLFTFHADICTLPDTEKQIKKQTALVELLKHKP
KATEEQLKTVMENFVAFVDKCCAADDKEACFAVEGPKLVVSTQTALA

Calculated Isotopically Averaged Molecular Weight of BSA = 66432.7 Da

Expected modifications: $Ru(CO)_2^+$ unit (*m/z* 157.9) and $Ru(CO)^+$ unit (*m/z* 129.9)

2.3 Liquid chromatography-mass spectrometry (LC-MS) under denaturing conditions

Liquid Chromatography-Mass Spectrometry (LC-MS) was performed on a Micromass Quattro API instrument (ESI-MS) coupled to a Waters Alliance 2795 HPLC using a MassPREP On-Line Desalting Cartridge 2.1 x 10 mm (Waters). Water:acetonitrile, 95:5 (solvent A) and acetonitrile (solvent B), with solvent A containing 0.1% formic acid, were used as the mobile phase at a flow rate of 0.3 mL/min. The gradient was programmed as follows: 95% A (0.5 minutes isocratic) to 80% B after 1.5 minutes, then isocratic for 1 minute, followed by 4 minutes to 95% A and finally isocratic for 6 minutes. The electrospray source was operated with a capillary voltage of 3.0 kV and a cone voltage of 20 V. Nitrogen was used as the nebulizer and desolvation gas at a total flow of 600 L/hr. Proteins typically elute on a single peak between 3 and 4.5 minutes using this method. For protein metallation analysis, the mass spectra corresponding to all protein in this peak were combined using MassLynx software (v. 4.0 from Waters). Mass spectra were calibrated using a calibration curve constructed from a minimum of 16 matched peaks from the multiply charged ion series of equine myoglobin (Sigma Aldrich), which was also obtained using the method described above. Total mass spectra were reconstructed from the ion series using the MaxEnt algorithm preinstalled on MassLynx software (v. 4.0 from Waters) according to manufacturer's instructions. The relative peak height that results from the reconstruction from total ion series is then used to calculate the relative amount of each protein and conjugation conversions. It is assumed that both modified and non-modified antibodies are ionized with similar efficiency. In the case of the metallation reactions reported, the excess of reagents could be removed by size exclusion chromatography and therefore did not interfere with LC-MS analysis.

2.4 Nondenaturing Nanoelectrospray Ionization Mass Spectrometry (Native Mass Spectrometry)

A 20 μL of BSA and BSA-$Ru^{II}(CO)_2$ samples were buffer exchanged into 200 mM ammonium acetate buffer (pH 7) using Micro Bio-Spin 6 columns (Bio-Rad). Mass spectra were acquired on a high-mass Q-TOF-type instrument Xevo G2-S (Waters, Manchester, UK). Mass spectrometry experiments were performed at a capillary voltage of 1500 V, cone voltage of 200 V and source offset voltage of 150 V. Spectra

were acquired in sensitivity mode that has resolution >22500 FWHM. MS data were processed using MassLynx V4.1 (Waters).

2.5 Cell culture

Caco-2 (ATCC; passage 10-22) and HeLa cells (ECACC; passage 10-22) were routinely grown in a humidified incubator at 37 ºC under 5% CO_2 and split twice a week before reaching confluence using 0.25% trypsin and 1% EDTA. Caco-2 cells were grown as monolayers using MEM GlutaMAX medium (Invitrogen, Life Technologies), supplemented with 20% heat-inactivated fetal bovine serum (FBS) (Gibco, Life Technologies), 1 mM sodium pyruvate, 200 units/mL penicillin and 200 μg/mL streptomycin (Gibco, Life Technologies). HeLa cells were grown on MEM GlutaMAX medium supplemented with 10% heat-inactivated fetal bovine serum (FBS), 10 mM HEPES (Gibco, Life Technologies), 200 units/mL penicillin and 200 μg/mL streptomycin (Gibco, Life Technologies).

2.6 Cell viability assay

HeLa cells were seeded in a 6 well-plate, at a density of 300 000 cells/well and incubated for 24 hours to allow for cell attachment. Cells were then treated with either BSA or BSA-$Ru^{II}(CO)_2$ and incubated for 24, 48 or 72 hours. Upon completion of the incubation, culture medium was removed, cells were washed with DPBS 1x (Gibco, Life Technologies) and incubated for 5 min, at 37 ºC, with TrypLExpress (Gibco, Life Technologies). Cells were washed with fresh medium and re-suspended in 1 mL complete medium. A 1:10 dilution in Trypan blue 0.4% (Gibco, Life Technologies) of this suspension was used to count cells using a Neubauer chamber, according to the manufacturer's instructions.

2.7 COP-1 fluorescence response to CO measured in buffered aqueous solution

COP-1 was synthesized according to the literature.[2] Fluorescence of COP-1 in the absence (negative control) or presence of 1.5 μM BSA-$Ru^{II}(CO)_2$ was determined on different time points using a fluorescence spectrometer, FLS920 (Edinburgh Instruments). A 1 μM solution of COP-1 was prepared in PBS pH 7.4 (without Calcium or Magnesium) from a 5 mM stock solution of COP-1 in DMSO. Experiments were performed at 37 ºC in 500 μL volume. Spectra were taken after the

addition of 1 μM COP-1 to 1.5 μM BSA-$Ru^{II}(CO)_2$ at 0, 10, 20, 30, 60, 90 and 120 min from 490 to 650 nm following excitation at λ_{ex} = 475 nm.

2.8 COP-1 fluorescence response by confocal microscopy imaging

Images were obtained using a Zeiss LSM 710 confocal Laser Point-Scanning Microscope with a 40X oil objective lens and a numerical aperture of 1.3. COP-1 was excited using an Argon Laser 488 nm and Hoescht 33342 was excited using a Diode Laser 405 nm and were read at green (λ_{em} 500-550 nm) and blue (λ_{em} 420-470 nm), respectively. Cells were imaged at 37 ºC and 5% CO_2 throughout the course of the experiment. $15x10^3$ HeLa cells were seeded in 8-chambered #1.0 Borosilicate coverglass (Lab-Tek), 2 days before the experiment. Culture conditions were the same used for routinely cell passage, using phenol red free MEM medium. Mean of total fluorescence intensity of treated versus untreated cells was compared using representative images of three independent experiments on a per cell basis. Statistical significant differences were analyzed after a two-way ANOVA. Data are presented in the graphs as mean of total fluorescent intensity ± SEM. CO release movie was assembled by acquiring images of a fixed field every two min for one hour.

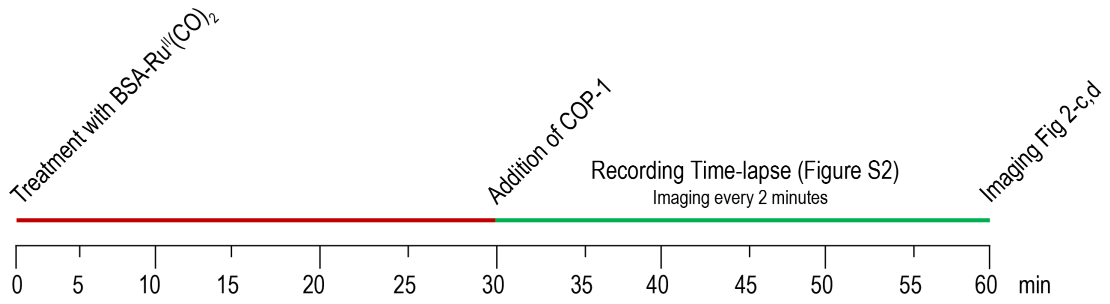

Illustration of the experimental procedure for CO selective imaging using COP-1 turn on fluorescent probe.

2.9 Chemokine modulation by CO release

Growth medium levels of the tested chemokines were quantified using Human IL-6, IL-8, IL-10 and TNF-α Mini ELISA Development Kits (Pepro-Tech, sensitivity range of 0.063 to 4 ng/mL) and revealed using TMB substrate reagent set (BD Biosciences) according to the manufacturer's protocol. The absorbance in each well was read at 450 nm by using a microplate reader (Infinite M200 microplate absorbance reader,

Tecan). Cells were plated at $2.5x10^5$ cell/well in 6 well plates. Two groups were tested: cells incubated with (treated) or without (untreated) 2 mL of 1.5 μM BSA-$Ru^{II}(CO)_2$, 48 hours after seeding. Supernatants were collected at 4, 12 and 24 hours post treatment. Statistical significant differences were analyzed after a two-way ANOVA post-hoc test using Bonferroni method. Data are presented in the graphs as mean ± SEM.

2.10 Bacterial strains and growth conditions

Escherichia coli K-12 MG1655 cells were grown aerobically in M9b medium,[3] at 37 ºC. The cultures were initiated by addition of exponential M9b-grown pre-cultures to an optical density at 600 nm (OD_{600}) of about 0.05, and grown until mid-exponential phase (OD_{600} of 0.3). At this stage, cells were left untreated or exposed to 50 μM CORM-3, 5 μM BSA-$Ru^{II}(CO)_2$ and 5 μM BSA, and growth was monitored hourly. The amount BSA-$Ru^{II}(CO)_2$ was calculated taking into account that each BSA molecule has 7 $Ru^{II}(CO)_2$ fragments attached. All solid compounds were freshly prepared as 50 mM stock solutions by dissolution in PBS pH 7.4 buffer.

2.11 In vivo CO biodistribution in tumor bearing mice

Tumor cell line

CT26 colon carcinoma cells (ATCC) were cultured with DMEM (Gibco, Life Technologies) supplemented with 10 % heat-inactivated fetal bovine serum (Gibco, Life Technologies) at 37 ºC with 5 % CO_2 and 95 % air in a humidified incubator.

Colon carcinoma tumor mouse model in immunodeficient mice

Tumor bearing mice were obtained by subcutaneous injection of CT26 colon carcinoma cells (5×10^7) into the left flank of 10-week-old female athymic BALB/c nu/nu mice (Charles River Laboratories). The tumors were allowed to grow for 14 days to a size of typically 200 mm^3. All animal experiments were carried out according to European regulations under project approved by the IMM Ethics Committee (AEC-2014-05-GB-Cancer).

In vivo biodistribution of CO

Biodistribution of CO released from BSA-$Ru^{II}(CO)_2$ in tumor bearing mice was performed according to the protocol described by Vreman and coworkers.[4] Female athymic BALB/c nu/nu mice (10-week-old – 3 mice per group) were administered intravenously with 3 mg/kg of BSA (control) or BSA-$Ru^{II}(CO)_2$. After 4 hours mice were sacrificed and perfused with 10 mL of cold PBS pH 7.4. Tissues were then collected, cut and weighted. 4 volumes of water (corresponding to 4 times the weight of the tissue) were added to the tissues, which were subsequently homogenized using a tissue tearor (Bio Spec Products). Aliquots of homogenate (30 μL) were then transferred into vials to which water (25 μL) and sulfosalicylic acid (Sigma-Aldrich, 5 μL, 30% wt/vol) were immediately added before the vials were closed with a gas tight cap. The vials were incubated on ice for 30 min. The released CO gas in the headspace of the vials was measured with a gas chromatograph (GC) equipped with a reducing-compound photometry detector (RCP) (Peak Laboratories, Mountain View). In this way it is possible to detect and quantify quantitatively gaseous CO at concentrations as low as 1-2 parts per billion (ppb). CO was calculated using a calibration curve prepared from CO standards. 3 independent measurements were performed for each mouse in the control and treated mice groups (3 mice per group).

2.12 Determination of COHb levels in blood

Samples of freshly collected blood 30 min and 4 hours after intravenous administration of BSA-$Ru^{II}(CO)_2$ were transferred to cuvettes. Levels of carboxyhemoglobin (COHb), oxyhemoglobin (O_2Hb), and methemoglobin (MetHb) were measured using an Avoximeter 4000 (ITC), whole blood CO-Oximeter. The results are presented as mean percentages of total hemoglobin species in circulation. 3 independent measurements were performed for each mouse in the control and treated mice groups (3 mice per group).

3. References

[1] C. T. Veros, N. J. Oldham, *Rapid Commun. Mass Spectrom.* **2007**, *21*, 3505-3510.

[2] B. W. Michel, A. R. Lippert, C. J. Chang, *J. Am. Chem. Soc.* **2012**, *134*, 15668-15671.

[3] P. N. ds Costa, M. Teixeira, L. M. Saraiva, *FEMS Microbiol. Lett.* **2003**, *218*, 385-393.

[4] H. J. Vreman, R. J. Wong, T. Kadotani, D. K. Stevenson, *Anal. Biochem.* **2005**, *341*, 280-289.
